# Removal of Pb(II) and Cd(II) from a Monometallic Contaminated Solution by Modified Biochar-Immobilized Bacterial Microspheres

**DOI:** 10.3390/molecules29194757

**Published:** 2024-10-08

**Authors:** Zaiquan Li, Xu Xiao, Tao Xu, Shiyu Chu, Hui Wang, Ke Jiang

**Affiliations:** 1 College of Eco-Environmental Engineering, Guizhou Minzu University, Guiyang 550025, China; lizaiquan2022@163.com (Z.L.); xiaoxu99024@163.com (X.X.); xutao001103@163.com (T.X.); chushiyu0829@163.com (S.C.); jiangke1@foxmail.com (K.J.); 2 Engineering Research Center of Green and Low-Carbon Technology for Plastic Application, Guizhou Minzu University, Guiyang 550025, China

**Keywords:** biochar, microbial immobilization, adsorption, lead and cadmium, contamination remediation

## Abstract

Lead (Pb) and cadmium (Cd) are toxic pollutants that are prevalent in wastewater and pose a serious threat to the natural environment. In this study, a new immobilized bacterial microsphere (CYB-SA) was prepared from corn stalk biochar and *Klebsiella grimontii* by sodium alginate encapsulation and vacuum freeze-drying technology. The removal effect of CYB-SA on Pb(II) and Cd(II) in a monometallic contaminated solution was studied. The results showed that the removal of Pb(II) and Cd(II) by CYB-SA was 99.14% and 83.35% at a dosage of 2.0 g/L and pH = 7, respectively, which was 10.77% and 18.58% higher than that of biochar alone. According to the Langmuir isotherm model, the maximum adsorption capacities of Pb(II) and Cd(II) by CYB-SA at 40 °C were 278.69 mg/g and 71.75 mg/g, respectively. A combination of the kinetic model, the isothermal adsorption model, scanning electron microscopy–energy dispersive X-ray spectroscopy (SEM-EDS), X-ray photoelectron spectroscopy (XPS) and Fourier-transform infrared spectroscopy (FTIR) analyses showed that the main adsorption mechanisms of CYB-SA encompass functional group complexation, ion exchange, electrostatic attraction and physical adsorption. The findings of this study offer practical and theoretical insights into the development of highly efficient adsorbents for heavy metals.

## 1. Introduction

The rapid advancement of the industrial and mining industry has led to an increasing influx of toxic heavy metals (HMs) into aquatic ecosystems [1] which pose a serious threat to aquatic ecosystems and can enter the human body through the food chain, with a significant risk to human health [2]. Heavy metal pollutants such as Pb(II) and Cd(II) are widely recognized as predominant contaminants in various industrial applications [3]. Prolonged exposure to Pb(II) and Cd(II) has been linked to neurological damage, reproductive system impairment, kidney diseases, and other adverse health effects [4,5]. Consequently, it is imperative to enhance measures for preventing and controlling water pollution caused by heavy metals like Pb(II) and Cd(II) [6]. Conventional techniques for removing Pb(II) and Cd(II) contaminants from water include chemical precipitation, ion exchange, membrane separation, and electrocoagulation [7]. However, these methods have limitations in practical implementation due to high energy consumption and low removal efficiencies [8]. Consequently, there is an urgent need for an efficient and environmentally friendly approach to address Pb(II) and Cd(II) contamination.

Biochar is a large-surface-area, porous, stable carbon-based substance with a variety of surface functional groups [9]. It has found widespread application in remediating heavy metal pollution [10]. China, being an agricultural nation, annually discards or burns a significant amount of corn straw in the open, resulting in environmental pollution. Consequently, producing biochar from corn straw offers a means of utilizing agricultural waste resources. Nonetheless, challenges such as recovery difficulty and limited adsorption efficiency arise during biochar application. Biochar modification can improve this situation to some extent. For example, Dong et al. utilized FeCl_3_ to modify biochar, resulting in magnetic biochar that demonstrated a substantially enhanced Pb(II) removal rate compared to the untreated biochar, along with excellent recovery capabilities [11].

Microbial remediation has gained attention due to its inherent advantages, including effectiveness, cost-effectiveness, and environmental friendliness [12]. When microorganisms are present at highly contaminated sites, they tend to adapt by converting toxic compounds into stable forms, thus preventing the formation of secondary contaminants [13]. In addition, microbial remediation facilitates the removal of low concentrations of contaminants that cannot be removed by physical or chemical methods. Pratyasha Pallavi et al. [14]. found that the adsorption efficiency of *Bacillus* sp. on Cr (VI) in water reached 89.14%. However, major problems including loss of microbial cells, reduced microbial proliferation and prolonged operation time have hindered their commercial and industrial application [15].

In this context, microbial immobilization technology has emerged as a promising solution for heavy metal remediation [16]. Microbiological immobilization technology utilizes physical or chemical methods to increase the concentration of free microorganisms by fixing them in a confined space, thereby sustaining high biological activity. Biochar is often chosen as a stationary material because of its large surface area and its ability to provide nutrients to microorganisms [17,18]. Furthermore, biochar has a variety of functional groups that contain oxygen, such as hydroxyl, carbonyl, and carboxyl groups [19]. These functional groups not only facilitate microbial colonization but also enable the immobilization of heavy metals [20]. Consequently, biochar is a perfect substrate for rendering microbes immobilized. According to research by Liu et al. biochar and bacteria that oxidize manganese greatly improve the removal of toxic heavy metals from water [21]. Compared to single applications, the removal efficiency for lead increased to 97.71% and for cadmium increased to 96.5%, approximately twice as effective as individual systems. Huang et al. utilized rice straw biochar immobilized with wax-like spore-forming bacterium RC-1, achieving a maximum adsorption capacity of 158.77 mg/g for Cd(II) [22]. Sodium alginate is a widely used natural linear anionic polysaccharide known for its excellent biocompatibility and biodegradability [23]. Encapsulating microorganisms with sodium alginate not only addresses the issue of recycling biochar but also enhances the reusability of the immobilized material [24]. Li et al. [25] achieved a removal rate of 100% for Pb(II) and 91.2% for Cd(II) using sodium alginate and polyvinyl alcohol encapsulated sulfate-reducing bacteria (SRB). In summary, immobilized bacterial conjugation can both reduce the stress of heavy metal environments on microorganisms and increase the adsorption capacity for heavy metals.

In this study, K_2_FeO_4_-modified corn stalk biochar served as a carrier, and *Klebsiella grimontii* was chosen as the immobilized bacteria to produce immobilized microbial composite materials via an adsorption-encapsulation method [26]. We examined the adsorption of this composite material on Pb(II) and Cd(II) and conducted comprehensive research on factors influencing adsorption, kinetics, isotherm models, and thermodynamics. The alterations in the composite material before and after adsorption were analyzed using SEM-EDS, FTIR and XPS to elucidate the process and mechanism of K_2_FeO_4_-modified corn stalk biochar immobilized *Klebsiella grimontii* for Pb(II) and Cd(II) removal. These results provide useful information for managing heavy metal pollution in aquatic bodies as well as theoretical ideas.

## 2. Results and Discussion

### 2.1. Comparison of Adsorption Effect of Different Adsorbents

**Figure 1:**
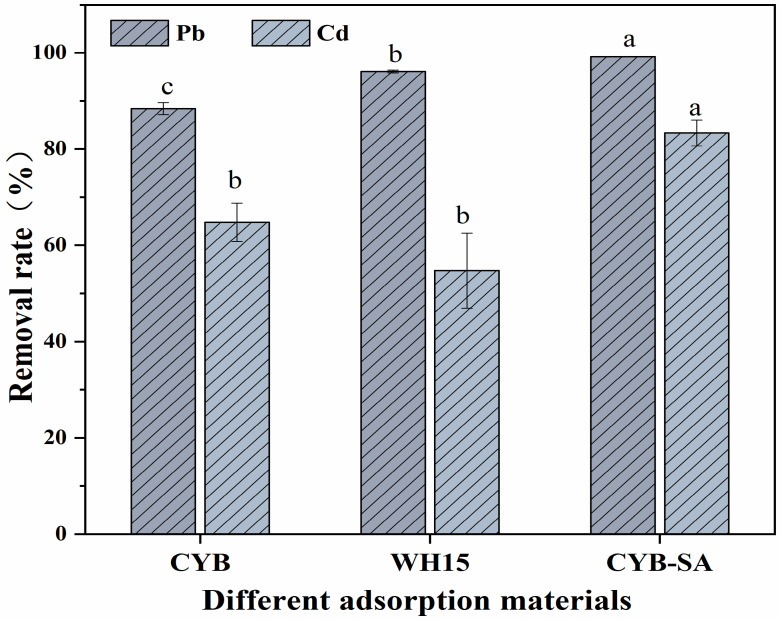
Effects of different adsorption materials on removal rates. (Note: Lowercase letters above the error bars indicate significant differences among different treatments (*p* < 0.05)).

The adsorption effects of different adsorption materials on Pb(II) and Cd(II) are shown in Figure 1. When the concentrations of Pb(II) and Cd(II) were 300 and 8 mg/L, respectively, the removal rates of Pb(II) and Cd(II) by CYB were 88.41% and 64.77%, while WH15 exhibited removal rates of 96.15% and 54.76% for Pb(II) and Cd(II). CYB-SA achieved the highest removal rates for Pb(II) and Cd(II). The removal rate of Pb(II) by CYB-SA was 99.18%, which was 10.77% and 3.03% higher than that of CYB and WH15, respectively. Similarly, CYB-SA demonstrated a Cd(II) removal rate of 83.35%, which exceeded those of CYB and WH15 by 18.58% and 28.59%, respectively.

For immobilized bacteria, the biochar carrier could effectively lower the concentrations of Pb(II) and Cd(II) in liquid medium at beginning, decreasing harm to developing bacteria within the immobilized pellet and consequently boosting bacterial activity and enhancing removal efficiency [27,28]. The immobilization of microbial cells on biochar has been shown in various studies to improve performance. For instance, when immobilized on biochar generated from the cyanobacterium D. flos-aquae, the metal ion-resistant bacterium *Proteus mirabilis* YC80 shown remarkable efficacy in eliminating Cr^6+^, exhibiting a 100% removal rate that was superior to that of free cell [29]. Zhou et al. showed the higher capacity of an immobilized *Ochrobactrum* sp. J023 to remove Pb^2+^ [30]. *Leclercia adecarboxylata* immobilized on rice hulls showed a high removal effectiveness of 93% for Pb^2+^, demonstrating the potential of biochar immobilized with microbial cells to address the removal of metal ions [31]. Therefore, when CYB-SA is in heavy metal stress, biochar has the function of protecting and fixing microorganisms [18].

### 2.2. Effect of Initial pH on Adsorption

**Figure 2:**
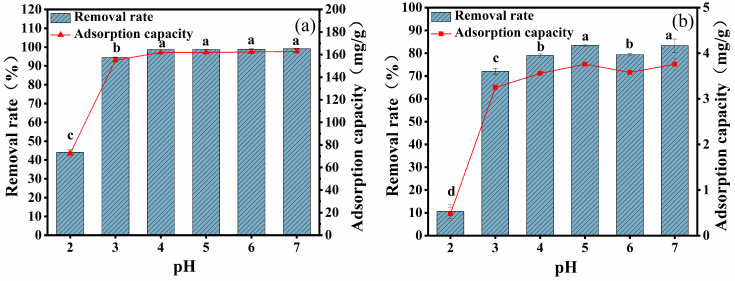
Effects of different pH on adsorption of Pb(II) (**a**) and Cd(II) (**b**). (Note: Lowercase letters above the error bars indicate significant differences among different treatments (*p* < 0.05)).

The changes in removal efficiency and adsorption capacity of CYB-SA for Pb(II) and Cd(II) under different initial solution pH conditions are depicted in Figure 2. With the increase in initial pH value, the removal efficiency and adsorption capacity for Pb(II) (Figure 2a) and Cd(II) (Figure 2b) first increase and then tend to stabilize. When pH = 7, CYB-SA demonstrates maximum removal efficiency for Pb(II) and Cd(II), with values reaching 99.14% and 162.80 mg/g for Pb(II), as well as 83.35% and 3.76 mg/g for Cd(II).

In the initial solution with pH = 2, CYB-SA exhibits lower removal efficiency and adsorption capacity for Pb(II) and Cd(II) due to competition between H^+^ ions and metal ions for adsorption sites [14]. The protonation of CYB-SA’s cell wall under acidic conditions results in a positive charge, which leads to the possibility of leading to electrostatic repulsion between CYB-SA and Pb(II), Cd(II) ions [32]. Moreover, changes in external pH directly impact microbial reproduction and metabolism, thereby influencing the adsorption effectiveness of CYB-SA towards Pb(II) and Cd(II). Compared with pH = 2 and 3, the removal rate and adsorption capacity were significantly increased and tended to be stable at 4 ≤ pH ≤ 7. As the pH increases, the OH^-^ concentration gradually rises in the solution. When H^+^ and OH^-^ are combined, the competition for adsorption sites on CYB-SA between H^+^ ions and Pb(II) and Cd(II) is diminished [33]. Simultaneously, deprotonation of functional groups weakens repulsive forces between charges, thus enhancing their adsorption capability. Based on an analysis of the adsorption performance, subsequent experiments were conducted at a controlled pH = 7.

### 2.3. Effect of Additive Amount

**Figure 3:**
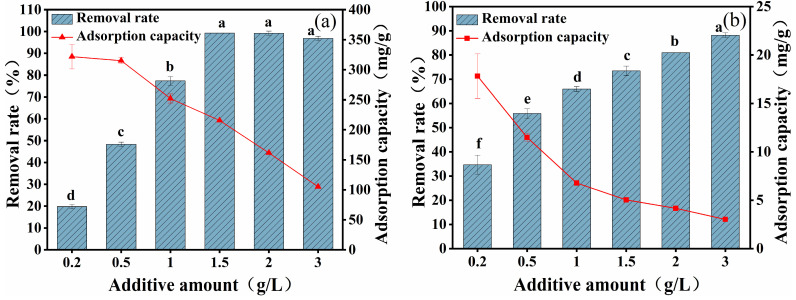
Effects of different CYB-SA addition amounts on adsorption of Pb(II) (**a**) and Cd(II) (**b**). (Note: Lowercase letters above the error bars indicate significant differences among different treatments (*p* < 0.05)).

The effects of different CYB-SA additions on the adsorption capacity of Pb(II) and Cd(II) are shown in Figure 3. As the CYB-SA dosage gradually increased, the removal efficiency of Pb(II) (Figure 3a) initially rose and then stabilized. Increasing the dosage from 0.2 g/L to 1.5 g/L resulted in the removal efficiency of Pb(II) rising from 19.75% to 99.28%. When the dosage increased further from 1.5 g/L to 3 g/L, the removal rate tended to be stable. Conversely, the removal efficiency of Cd(II) (Figure 3b) showed a continuous increasing trend. Increasing the dosage from 0.2 g/L to 3 g/L resulted in the removal efficiency of Cd(II) increasing from 34.63% to 88.18%. Simultaneously, the continuous increase in CYB-SA dosage resulted in a gradual decrease in the adsorption capacity for both Pb(II) and Cd(II).

Keeping the initial concentrations of Pb(II) and Cd(II) in the solution unchanged at 300 and 8 mg/L, respectively, increasing the dose of CYB-SA can increase the effective absorption point and microbial content provided by the adsorbent. As a result, the rate of removal rose while the amount of Pb(II) and Cd(II) adsorbed gradually reduced [34]. By integrating adsorption efficiency with economic analysis, the optimal CYB-SA dosage for subsequent experiments was determined to be 2 g/L.

### 2.4. Adsorption Kinetics

**Figure 4:**
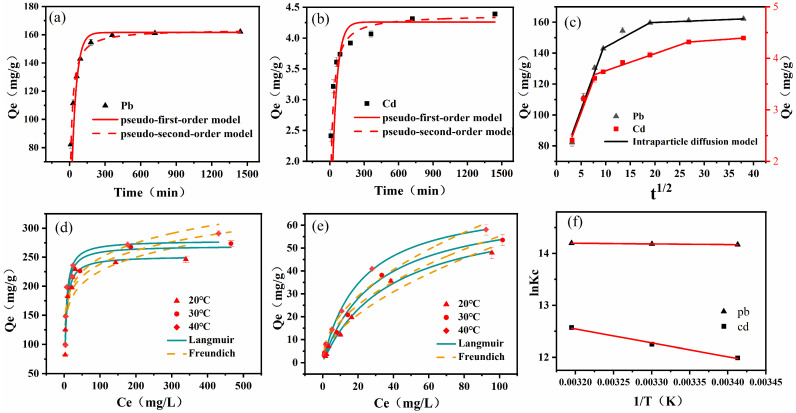
Adsorption kinetics of Pb(II) (**a**) and Cd(II) (**b**), intraparticle diffusion (**c**), the isothermal adsorption process of Pb(II) (**d**) and Cd(II) (**e**) and adsorption thermodynamics (**f**).

Pseudo-first-order and pseudo-second-order kinetic models were used to match the experimental data. The fitting graphs and associated parameters are shown in Figure 4a,b and Table 1. The adsorption of Pb(II) and Cd(II) by CYB-SA initially exhibited a rapid increase, followed by a gradual slowdown until reaching adsorption equilibrium. Hence, the adsorption process can be categorized into two stages: rapid adsorption and slow adsorption. In the initial 0 to 90 min for rapid adsorption phase, the adsorption capacities of Pb(II) and Cd(II) surged from 82.29 mg/g and 2.41 mg/g to approximately 143.00 mg/g and 3.74 mg/g, representing about 88.20% and 85.19% of the equilibrium adsorption capacities. Subsequently, the period from 90 to 1440 min indicates the slow adsorption stage, wherein Pb(II) and Cd(II) approached equilibrium at 360 and 720 min. Additionally, the R^2^ values for the pseudo-second-order kinetic fitting results of Pb(II) and Cd(II), as shown in Table 1, were 0.9851 and 0.9529, respectively, surpassing the pseudo-first-order kinetic fitting results, which stood at 0.8786 and 0.7345. Furthermore, the equilibrium adsorption capacities of Pb(II) and Cd(II) calculated by the pseudo-first-order and pseudo-second-order kinetic models closely aligned with the experimental results, with values of 161.60 mg/g and 4.26 mg/g, and 163.77 mg/g and 4.38 mg/g. Consequently, considering the R^2^ values, it can be concluded that the pseudo-second-order kinetic model provides a more accurate description of the adsorption process of CYB-SA for Pb(II) and Cd(II), primarily governed by chemical adsorption [7,35].

To further understand the diffusion mechanism controlling the adsorption process of Pb(II) and Cd(II) by CYB-SA, the intraparticle diffusion model was employed to fit the adsorption results. The fitting graph along with the corresponding parameters is presented in Figure 4c, while Table 2 provides detailed information. The model of intraparticle diffusion using the diffusion model can be divided into three main stages [36]. The first stage is surface diffusion, in which heavy metal ions diffuse from the aqueous phase to the outer surface of CYB-SA. The electrostatic attraction between heavy metal ions and adsorption sites plays a major role in this stage. The second stage is known as intraparticle diffusion adsorption, in which heavy metal ions enter the inside hole from the external surface of CYB-SA and diffuse to the inner surface of CYB-SA at a slow speed. The third stage is the adsorption saturation stage, in which heavy metal ions are adsorbed by the action points on CYB-SA and the reaction of adsorption reaches a stable state. Table 2 reveals that C_1_, C_2_, and C_3_ are all non-zero, indicating that intraparticle diffusion alone does not entirely govern the adsorption process [37]. Instead, multiple adsorption mechanisms collaboratively influence the entire process.

### 2.5. Adsorption Isotherms

Adsorption isotherms at 20 °C, 30 °C and 40 °C were fitted using the Langmuir and Freundlich models. The fitting curves and characteristic parameters are displayed in Figure 4d,e, while Table 3 provides detailed data. The obtained R^2^ values suggest that the Langmuir model provides a better fit to the isothermal adsorption data compared to the Freundlich model, indicating monolayer chemisorption of Pb(II) and Cd(II) by CYB-SA. The Langmuir model calculates the maximum adsorption capacities of CYB-SA for Pb(II) and Cd(II) as 278.69 mg/g and 71.75 mg/g. The CYB-SA exhibited superior adsorption performance. In addition, in comparison to other studies (Table 4), CYB-SA also has excellent adsorption properties. The addition of biochar mitigated the toxic effects of heavy metals on microorganisms. Table 3 indicates that the R_L_ values of Pb(II) and Cd(II) at different temperatures are in the range of 0.0043–0.0234 and 0.0977–0.8661 (between 0–1), indicating that the adsorption process can proceed smoothly [38]. The 1/n values in the Freundlich model, ranging from 0 to 1, suggest that both Pb(II) and Cd(II) are readily adsorbed by CYB-SA, primarily through chemical adsorption [39].

### 2.6. Adsorption Thermodynamics

To investigate the thermodynamic properties of adsorption, the thermodynamic parameters (ΔH^θ^, ΔS^θ^ and ΔG^θ^) were calculated by plotting the relationship between lnKc and 1/T and determining the slope and intercept of the line (Figure 4f). As shown in Table 5, ΔG^θ^ exhibited negative values at different temperatures, while both ΔH^θ^ and ΔS^θ^ showed positive values. The negative value of ΔG^θ^ indicates the spontaneous occurrence of Pb(II) and Cd(II) adsorption on CYB-SA. Moreover, an increase in temperature led to a higher absolute value of ΔG^θ^, indicating enhanced spontaneity and favorability for adsorption. The positive value of ΔH^θ^ suggests that the adsorption process involving Pb(II) and Cd(II) by CYB-SA is endothermic; thus, elevated temperatures are advantageous for heavy metal adsorption, as supported by experimental findings. Furthermore, when ΔS^θ^ is positive, it indicates that the adsorbent and solution undergo structural alterations through the adsorption process, which increases the degree of unpredictability at their interface [48].

### 2.7. Post-Adsorption Characterization Results and Adsorption Mechanism Analysis

**Figure 5:**
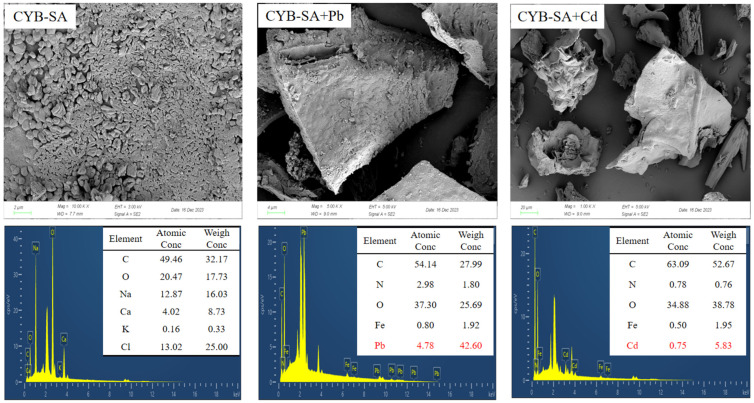
SEM-EDS diagram before and after CYB-SA adsorption.

The electron microscopy energy spectrum images of CYB-SA before and after adsorption are presented in Figure 5. Prior to the adsorption of Pb(II) and Cd(II), numerous *Klebsiella grimontii* WH15 microorganisms can be observed on the surface of CYB-SA. These microorganisms manifest as short rod-like structures with wrinkled features, suggesting their successful loading onto the biochar. The EDS data revealed that Pb(II) and Cd(II) were detected after adsorption, which confirmed that the two ions were successfully adsorbed on CYB-SA.

**Figure 6:**
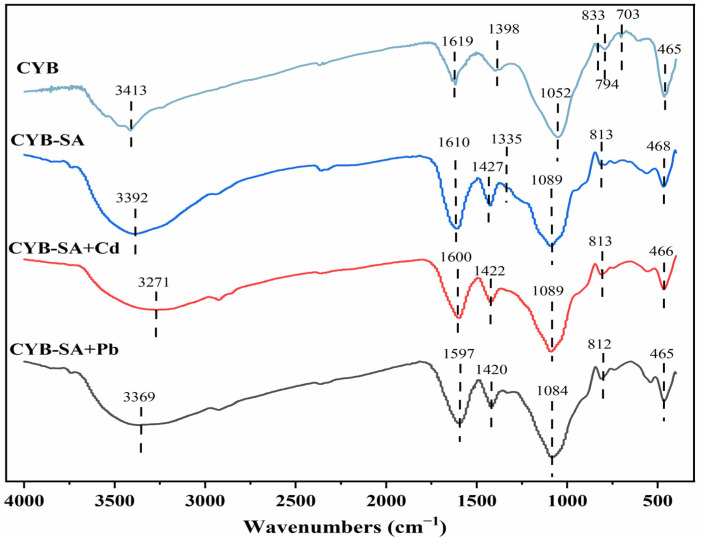
FTIR diagram before and after CYB-SA adsorption.

The FTIR spectrum is presented in Figure 6. The peak of CYB at 1052 cm^−1^ is caused by C-O stretch vibrations. The presence of the C=C stretching vibration at 1398 cm^−1^ signifies the presence of a stable aromatic framework on the carbon surface, which promotes cell adhesion and proliferation [49]. The characteristic peak at 3392 cm^−1^ of CYB-SA is attributed to the -OH stretching vibration. The characteristic peak at 1427 cm^−1^ is caused by the C=C stretching of aromatic ring [50]; The characteristic peak at 1089 cm^−1^ is caused by the stretching vibration of C-O-C and C-C [51]; Additionally, the aromatic C-H bending vibration is what causes the absorption peak at 813 cm^−1^ [52]. After adsorption of Pb(II) and Cd(II), the position of -OH at 3392 cm^−1^ shifted compared to CYB-SA obviously, indicating that -OH may act as an electron donor to form complexes with Pb(II) and Cd(II) [53]. The movement from 1610 cm^−1^ to 1600, 1597 cm^−1^ is attributed to the vibrational stretching of bacterial surface proteins or amine C=O [30]. The results showed that CYB-SA showed functional group changes compared with CYB after fixing WH15 [14]. The aromatic ring C=C at 1427 cm^−1^ moved to the vicinity of 1422 and 1420 cm^−1^, indicating that C-X functional groups may participate in the adsorption process [54]. The spectra can be compared to observe that while the number of peaks before and after CYB-SA adsorption did not change significantly, their intensity and position did. These changes can be attributed to the interaction between the heavy metals and the functional groups on CYB-SA, such as surface complexation or ion exchange, which forms complexes [55].

**Figure 7:**
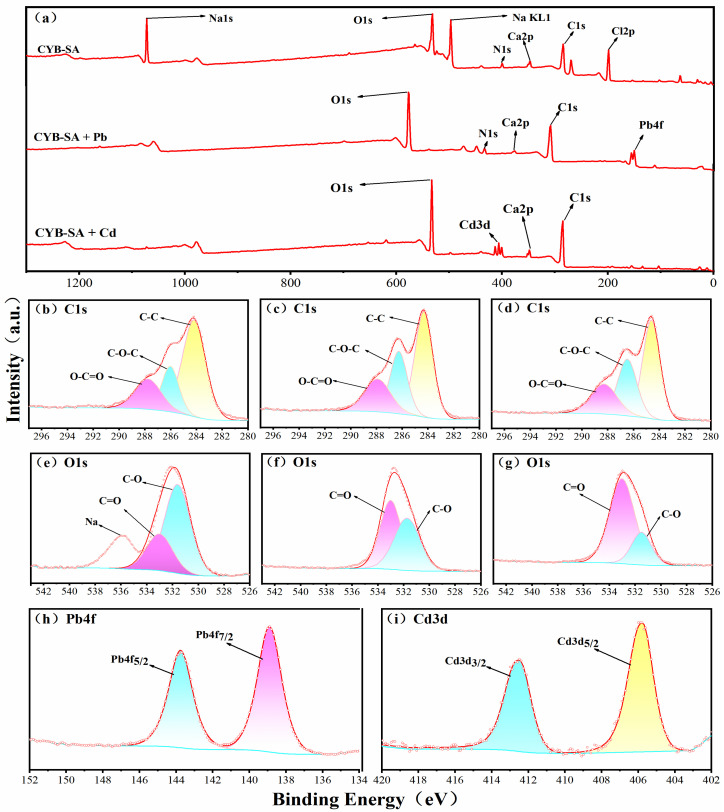
XPS spectra before and after adsorption of Pb(II), and Cd(II) by CYB-SA, Survey (**a**), C1s-CYB-SA (**b**), C1s-Pb(II) (**c**), C1s-Cd(II) (**d**), O1s-CYB-SA (**e**), O1s-Pb(II) (**f**), O1s-Cd(II) (**g**), Pb(II) (**h**), and Cd(II) (**i**).

The XPS spectrum of CYB-SA before and after adsorption of Pb(II) and Cd(II) is shown in Figure 7. It can be observed from Figure 7a that the characteristic peaks of Pb4f and Cd3d appear in the full spectrum after adsorption of Pb(II) and Cd(II), indicating that CYB-SA successfully achieved the adsorption of Pb(II) and Cd(II).

The C1s spectral peak of CYB-SA before and after Pb(II) and Cd(II) adsorption is depict in Figure 7b–d, which are mainly divided into three parts. The peaks before adsorption are located at 284.21, 286.05 and 287.78 eV, corresponding to C-C, C-O-C and O-C=O bonds. After adsorption, the C-C peak decreases from 58.67% to 49.84% and 48.52%, while the C-O-C peak increases from 20.47% to 23.70% and 24.14%, and the O-C=O peak also increases from 20.86% to 26.47% and 27.34%. The development of hydroxyl/carbonyl-Pb^2+^/Cd^2+^ complexes may be the cause of these alterations [56,57].

The O1s spectra before and after CYB-SA adsorption are shown in Figure 7e–g, where the spectral peaks are mainly divided into two peaks. Before adsorption, 531.62 eV and 532.77 eV correspond to C-O and C=O bonds, respectively. After adsorption, the C-O peak decreased from 76.93% to 48.25% and 23.39%, while the C=O peak increased from 23.07% to 51.75% and 76.61%. The change in peak area ratio also indicates the presence of ion exchange during the fixation of heavy metals [47]. The characteristic double peaks of Pb4f are shown in Figure 7h, and the spectral peaks are concentrated at 138.89 eV (Pb4f_7/2_) and 143.75 eV (Pb4f_5/2_). The separation of the Pb4f_7/2_ and Pb4f_5/2_ double peaks by 4.86 eV indicates the existence of Pb-O bonds [7,58]. The characteristic peaks of Cd3d are shown in Figure 7i, and the spectral peaks are concentrated at 405.78 eV (Cd3d_5/2_) and 412.49 eV (Cd3d_3/2_). This means that the Cd(II) adsorbed on the surface of CYB-SA exists in the form of Cd-S [59], Cd-O [35] or Cd(II)-π [60].

The characteristic peaks of FTIR did not have obvious changes from those before adsorption, but the element Pb(II)/Cd(II) appeared in the EDS and XPS pattern. It was speculated that the adsorption mechanism of Pb(II)/Cd(II) adsorption by CYB-SA was mainly surface physical adsorption and pore interception, which agreed with the results of the isothermal adsorption model and the kinetic adsorption model [6]. FTIR and XPS analysis revealed that the characteristic peaks of the functional groups on the surface of CYB-SA adsorbed Pb(II)/Cd(II) were obvious changed, and it was speculated that functional group complexation is involved in the reaction [30]. The contents of Na after adsorption are lower than those before adsorption (Figure 7a), indicating that Na participated in the ion exchange and precipitation of Pb(II)/Cd(II) [46]. The adsorption experiments at different pH also show that there is electrostatic attraction between CYB-SA and heavy metal ions. To summarize, CYB-SA adsorbs Pb(II) and Cd(II) in solution by physical adsorption, complexation with functional groups containing oxygen, and ion exchange.

## 3. Materials and Methods

### 3.1. Experimental Materials

The corn stalk biochar (YB) and K_2_FeO_4_ were mixed and ground for 5 min at a mass ratio of 1:4, followed by pyrolysis at 500 °C in a Muffle furnace for 2 h. After cooling to room temperature, the mixture was washed with ultra-pure water until colorless. Finally, it was dried for 24 h at 60 °C to obtain modified corn stalk biochar (CYB) loaded with K_2_FeO_4_.

The strain *Klebsiella grimontii* WH15 was isolated from soil contaminated by lead and zinc mines in Weining County, Guizhou Province, China.

The reagents used in this experiment were sodium alginate (SA), calcium chloride anhydrous (CaCl_2_5H_2_O), sodium chloride (NaCl), lead chloride (PbCl_2_), cadmium nitrate tetrahydrate (Cd(NO_3_)_2_·4H_2_O), sodium hydroxide (NaOH), hydrochloric acid (HCl), beef extract, peptone. The above chemicals were purchased from Shanghai Macklin Biochemical Technology Co., Ltd. (Shanghai, China). The corn stalk biochar purchased from Henan Lize Biotechnology Company. LB medium includes 10 g/L peptone, 10 g/L NaCl, and 5 g/L beef extract, the sterilization process is conducted at 121 °C for 20 min.

### 3.2. Preparation of CYB-SA

The bacteria WH15 were inoculated at a volume of 2% (*v*/*v*) into 50 mL of LB liquid medium and incubated at 30 °C with oscillation at 150 rpm until under constant temperature reaching an optical density of 1.0–1.2 at 600 nm. Thoroughly combine the bacteria with a sterile SA solution of 4% (*w*/*v*). Then, the sterilized 1 g of CYB was mixed with 40 mL of bacteria and further oscillated at 30 °C and 150 rpm for 12 h. A 4% (*w*/*v*) SA sterilized solution was added and thoroughly mixed. The resulting mixture was added dropwise to a 2% (*w*/*v*) CaCl_2_ solution using a 5 mL syringe, allowing for cross-linking and hardening under refrigeration at 4 °C for up to 12 h. Biochar loaded bacterial microspheres composites were synthesized, and designated as CYB-SA. After undergoing three rounds of ultra-pure water washing, the final product was vacuum freeze-dried for 48 h and then kept at 4 °C.

### 3.3. Batch Experiments

#### 3.3.1. Adsorption Experiments with Different Adsorbents

The objective of this study is to investigate the adsorption capacity of various adsorbent materials for Pb(II)/Cd(II). Strain WH15 were pre-cultured for 24 h in LB medium at 30 °C and 180 rpm on a shaker. Then, cells were inoculated into a 50 mL LB liquid culture medium containing Pb(II)/Cd(II) (300/8 mg/L) at a 2% volume ratio. 0.1 g of CYB/CYB-SA were added to conical flasks, containing Pb(II)/Cd(II) at concentrations of 300/8 mg/L, respectively. The solution was adjusted to a pH value of 7 and subjected to oscillation at a temperature of 30 °C for a duration of 24 h. Once the sorption equilibrium has been reached, the supernatant was filtered through a 0.45 μm filter membrane, and an atomic absorption spectrophotometer was used to measure the Pb(II)/Cd(II) concentration. The adsorption capacity (Equation (1)) and removal rate (Equation (2)) of Pb(II)/Cd(II) calculated by:

(1) $$Q_{e}=\frac{(c_{0}-c_{e})V}{m}$$

(2) $$R_{e}=\frac{c_{0}-c_{e}}{c_{0}}\times 100\%$$

where Q_e_ is the equilibrium adsorption capacity (mg/g); C_0_ and C_e_ are the initial and equilibrium HMs concentrations (mg/L); V is the solution volume (L); m is the amount of CYB-SA (g); R_e_ is the removal rate (%).

#### 3.3.2. Adsorption Experiments at Different CYB-SA Addition Amounts and pH Values

In order to investigate the effect of different addition amounts on adsorption efficiency. Take 50 mL of Pb(II)/Cd(II) (300/8 mg/L) in a 150 mL conical flask, and add CYB-SA at ratios of 0.2, 0.5, 1.0, 1.5, 2.0 and 3 g/L, respectively, with shaking under constant temperature at 30 °C and 150 rpm for 24 h. The impact of pH on the removal of Pb(II)/Cd(II) by CYB-SA was investigated. Initial pH of the Pb(II)/Cd(II) (300/8 mg/L) solutions was adjusted to a range of 2–7 using 0.1 mol/L NaOH and 0.1 mol/L HCl, supplemented with 0.1 g CYB-SA and followed by oscillation at 30 °C for 24 h. After adsorption equilibrium, the supernatant was filtered by 0.45 μm filter membrane, and the concentrations of Pb(II)/Cd(II) were determined by atomic absorption spectrophotometer.

#### 3.3.3. The Adsorption Kinetics Model

To gain further insights into the adsorption mechanism and the rate-limiting steps, the concentrations of Pb(II)/Cd(II) in water was adjusted to 300/8 mg/L, and 0.1 g CYB-SA was added to perform adsorption kinetics experiments. Subsequently, samples were collected at various time intervals: 10, 30, 60, 90, 180, 360, 720 and 1440 min to obtain kinetic data. The kinetic data were fitted by pseudo-first-order (Equation (3)), pseudo-second-order (Equation (4)), and intraparticle diffusion models (Equation (5)) [61]:

(3) $$Q_{t}=Q_{e}(1-e^{-k_{1}t})$$

(4) $$Q_{t}=\frac{k_{2}Q_{e}^{2}t}{1+{Q_{e}k}_{2}t}$$

(5) $$Q_{t}=k_{p}t^{0.5}+C$$

where Q_e_ is the equilibrium adsorption capacity (mg/g); k_1_ (h^−1^) and k_2_ (g/(mg·h^−1^)) are the rate constants of pseudo-first-order and pseudo-second-order, respectively. k_p_ is the rate constant of the diffusion model (g/(mg·h^−0.5^)). C is a constant that is correlated with the thickness of the boundary layer (mg/g).

#### 3.3.4. Adsorption Isotherms and Thermodynamics Model

Initial concentrations of Pb(II) and Cd(II) were set at 200, 300, 400, 450, 500, 700, 1000 mg/L and 6, 8, 12, 30, 50, 100, 200 mg/L, respectively. Add 0.1 g CYB-SA to the solution. Adsorption isotherm and thermodynamic experiments were carried out at 20, 30, and 40 °C. The isotherm data were fitted using Langmuir and Freundlich models (Equations (6) and (7)). The dimensionless parameter R_L_ was employed to assess the ease of the adsorption reaction (Equation (8)):

(6) $$Q_{e}=\frac{K_{L}Q_{m}c_{e}}{1+K_{L}c_{e}}$$

(7) $$Q_{e}=K_{F}{c_{e}}^{\frac{1}{n}}$$

(8) $$R_{L}=\frac{1}{1+K_{L}c_{0}}$$

where Q_e_ and Q_m_ are the equilibrium adsorption capacity and the maximum adsorption capacity (mg/g), respectively. C_0_ and C_e_ are the initial and equilibrium HMs concentrations (mg/L); K_L_ is the Langmuir model constant (L/mg); k_F_ ((mg/g)·(mg/L)^1/n^) and n are Freundlich constants related to adsorption capacity and adsorption strength, respectively. R_L_ is the equilibrium constant used to determine the ease or difficulty of reaction progress.

Based on the analysis of adsorption isotherm, Gibbs free energy ΔG^θ^ (Equations (9) and (11)), enthalpy ΔH^θ^, and entropy changes ΔS^θ^ (Equation (10)) were examined to provide further insights into the energy involvement and spontaneous reaction of CYB-SA in removing Pb(II)/Cd(II) [62,63,64]:

(9) $$\Delta G^{\theta }=-RTlnK_{d}$$

(10) $$lnK_{d}=\frac{\Delta S^{\theta }}{R}-\frac{\Delta H^{\theta }}{RT}$$

(11) $$K_{d}=K_{L}\times 55.5\times 1000\times M_{a}$$

where ΔG^θ^ is Gibbs free energy (kJ/mol); ΔH^θ^ is standard molar enthalpy change (kJ/mol); ΔS^θ^ for standard Moore office (J/mol·K); R is the gas constant (8.314 J/(mol·K)); T is the temperature (K); K_d_ is adsorption equilibrium constant; M_a_ is the molecular weight of the adsorbed material.

### 3.4. Material Characterization

The infrared spectrogram analysis of samples (CYB-SA) was carried out with a Fourier transform infrared spectrometer (FTIR, Thermo Scientific Nicolet 6700, Waltham, MA, USA). The wavelength range was 400~4500 cm^−1^. The element binding state and surface composition of CYB-SA before and after adsorption were determined by X-ray photoelectron spectroscopy (XPS, Thermo Scientific K-Alpha). The surface morphology and element composition were observed with a scanning electron microscope (SEM, ZEISS Gemini SEM 300, Oberkochen, Germany) equipped with energy dispersive X-ray spectroscopy (EDS, OXFORD Xplore 30, Oxford, UK). The concentration of heavy metals was determined by a flame atomic absorption spectrophotometer (HITACHIZA3300, Tokyo, Japan).

## 4. Conclusions

In this study, the heavy metal-resistant bacteria *Klebsiella grimontii* WH15 was successfully immobilized on K_2_FeO_4_-modified corn straw biochar. When the concentrations of Pb(II) and Cd(II) were 300 and 8 mg/L, respectively, the removal rate of Pb(II) by CYB-SA was 10.77% and 3.03% higher than that of CYB and WH15, and the removal rate of Cd(II) was 18.58% and 28.59% higher than that of CYB and WH15, respectively. The adsorption capacity of CYB-SA for Pb(II) and Cd(II) reached 278.69 and 71.75 mg/g, demonstrating superior adsorption performance compared to free bacteria and pristine biochar. The adsorption kinetics and isotherm properties of CYB-SA to Pb(II) and Cd(II) are better described by the pseudo-second-order kinetic model and the Langmuir isotherm model. In addition, the thermodynamic parameter calculation showed that ΔH^θ^ and ΔS^θ^ were positive values, while ΔG^θ^ was negative, further proving that the adsorption was a thermodynamic reaction process of the monomolecular layer dominated by chemical adsorption. The results of SEM-EDS, FTIR, XPS and batch experiments show that the adsorption of Pb(II) and Cd(II) on CYB-SA is mainly the interaction of functional group complexation, ion exchange, electrostatic attraction and physical adsorption. These findings indicate that the use of bacteria resistant to heavy metals that have been immobilized on biochar for the remediation of heavy metal pollution is a viable and workable approach, and they offer a useful theoretical structure for the remediation of pollution caused by heavy metals.

## Figures and Tables

**Table 1:** Kinetic parameters for Pb(II) and Cd(II) adsorption on CYB-SA were obtained from the pseudo-first-order and pseudo-second-order models.

HMs	Experimental Value	Pseudo-First-Order Model			Pseudo-Second-Order Model		
	Q_max_ (mg/g)	Q_e_ (mg/g)	K_1_	R^2^	Q_e_ (mg/g)	K_2_	R^2^
Pb	162.13	161.60	0.0252	0.8786	163.77	4.7968	0.9851
Cd	4.39	4.26	0.0236	0.7345	4.38	0.0150	0.9529

**Table 2:** Intraparticle diffusion models fitting parameters.

HMs	K_p1_ (g/(mg·h^−0.5^))	C_1_ (mg/g)	R_1_^2^	K_p2_ (g/(mg·h^−0.5^))	C_2_ (mg/g)	R_2_^2^	K_p3_ (g/(mg·h^−0.5^))	C_3_ (mg/g)	R_3_^2^
Pb	8.80	59.71	0.9854	1.75	126.50	0.9967	0.13	157.24	0.9889
Cd	0.26	1.66	0.9731	0.03	3.42	0.9963	0.01	4.13	1.0000

**Table 3:** Fitting parameters of the isothermal adsorption model.

HMs	T	Langmuir				Freundlich		
		Q_m_ (mg/g)	K_L_	R_L_	R^2^	K_F_	1/n	R^2^
Pb	20 °C	252.17	0.2286	0.0044—0.0214	0.9584	114.9827	0.1466	0.7554
	30 °C	269.56	0.2311	0.0043—0.0212	0.9341	126.9482	0.1365	0.8211
	40 °C	278.69	0.2351	0.0042—0.0208	0.9129	128.0765	0.1439	0.7859
Cd	20 °C	68.18	0.0258	0.1625—0.8661	0.9945	3.8819	0.5619	0.9711
	30 °C	69.59	0.0335	0.1299—0.8327	0.9860	5.8029	0.4890	0.9753
	40 °C	71.75	0.0462	0.0977—0.7830	0.9986	6.7549	0.4860	0.9668

**Table 4:** The adsorption capacity of Pb(II) and Cd(II) by various adsorbents.

Adsorbents	Contaminants	Qm (mg/g)	References
Wheat heat straw and natural hematite-magnetic biochar	Pb(II)	196.91	[40]
Magnetic biochar-microbe biochemical composite	Cd(II)	25.04	[41]
Sulfide-modified magnetic pinecone-derived hydrochar	Pb(II), Cd(II)	49.33, 62.49	[42]
Wood ear mushroom sticks biochar	Pb(II), Cd(II)	234.20, 46.16	[43]
MnOx-impregnated on peanut shells derived biochar	Pb(II), Cd(II)	164.59, 36.77	[44]
White tea residue-magnetic biochar	Pb(II), Cd(II)	81.60, 38.60	[45]
Calcium alginate-nZVI-biochar	Pb(II), Cd(II)	247.99, 47.27	[46]
Enterobacter bugandensis immobilized by rice straw biochar	Pb(II)	250.10	[47]
Modified corn stalk biochar-supported bacterial microspheres	Pb(II), Cd(II)	278.69, 71.75	This study

**Table 5:** Thermodynamic characteristic parameters.

HMs	ΔH^θ^ (kJ/mol)	ΔS^θ^ (J/mol)	ΔG^θ^ (kJ/mol)		
			20 °C	30 °C	40 °C
Pb	1.07	121.44	−34.52	−35.72	−36.95
Cd	22.18	175.26	−29.20	−30.86	−32.71

## Data Availability

The data that support the findings of this study are available from the corresponding author upon request.
